# Editorial: Mechanism of neural oscillations and their relationship with multiple cognitive functions and mental disorders

**DOI:** 10.3389/fnins.2024.1543731

**Published:** 2025-01-07

**Authors:** Chuanliang Han

**Affiliations:** School of Biomedical Sciences and Gerald Choa Neuroscience Institute, The Chinese University of Hong Kong, Shatin, Hong Kong SAR, China

**Keywords:** neural oscillation, cognitive function, mental disorder, neuroelectrophysiology, power spectrum

Neural oscillations serve as a crucial biological bridge between the micro and macro levels of brain activity (Han et al., 2021a; Buzsáki and Vöröslakos, 2023). These oscillations play key roles in various functions (Han et al., 2021c; Lin et al., 2024). Moreover, abnormal neural oscillations are recognized as potentially influential factors in the development of a wide range of mental disorders (Han et al., 2022a; Wang et al., 2024; Zikereya et al., 2024). However, our understanding of the mechanisms underlying neural oscillations and their functional roles in different cognitive functions and mental disorders remains limited. Through this Research Topic, 10 related papers in the field are published, including research and review papers in different aspects. Although we have not yet been able to fully answer the question of neural oscillation mechanisms, these studies have nonetheless made significant progress, advancing step by step toward this goal.

Mood disorders are increasingly receiving attention from researchers [major depression diorder (MDD) or bipolar disorder (BD)]. In this Research Topic, there are seven papers involving mood disorders, which provide deeper insights into our understanding of their oscillatory mechanisms. Liu et al. analyzed EEG signals to enhance the diagnosis of MDD. By examining resting-state EEG under both eyes-closed and eyes-open conditions, asymmetries in band power between hemispheres were key predictors of MDD. This multi-region, multi-condition approach improves diagnostic accuracy for MDD, suggesting EEG could be a valuable tool for objective MDD diagnosis and treatment planning. Zhang et al. explores how alpha oscillations in the medial occipital cortex (MOC) mediate the relationship between depression severity and suicide risk in MDD. It found that higher depression severity increases suicide risk, while greater MOC alpha power serves as a protective factor. Specifically, MOC alpha power partially mediates the effect of depression severity on suicide risk, with a decrease in alpha power linked to higher suicide risk. Wang et al. investigates the alterations in EEG functional connectivity (FC) in individuals with MDD compared to healthy controls (HCs). Using resting-state EEG and phase locking value (PLV) analysis across five frequency bands (theta, alpha, and beta), the study found lower FC in MDD patients in certain brain regions, notably in the right temporal-left occipital cortex. No significant correlation was found between FC differences and depression severity. The study suggests EEG-based FC could be a promising tool for objective MDD diagnosis. Su et al. reviews the latest resting-state EEG (rsEEG) findings in BD, highlighting abnormal oscillations across multiple frequency bands (delta, theta, beta, and gamma), often marked by increased power. These abnormalities suggest widespread neural dysfunction. However, alpha oscillations showed more variability, potentially influenced by disease severity and sample diversity. The review stresses the importance of standardized experimental designs, considering factors like gender, age, medication, and methodology. These insights could help improve BD diagnosis and treatment.

Moreover, animal experiments with gamma-band activities also contribute a lot in this field (Han et al., 2021b, 2022b), Bergosh et al. explores the use of ketamine (KET) and medial prefrontal cortex deep brain stimulation (mPFC DBS) as treatments for depression in a rodent model. It examines how electrophysiological biomarkers, such as spectral parameters and sample entropy in local field potentials (LFP), correlate with antidepressant-like behaviors. Results suggest that changes in theta and gamma activity, along with sample entropy, may serve as biomarkers for depression severity and treatment efficacy, supporting both KET and mPFC DBS as potential therapeutic strategies. Neuhäusel and Gerevich investigates the sex-specific effects of the NMDA receptor antagonist MK-801 on hippocampal gamma oscillations in rats. It finds that female rats are more sensitive to MK-801, showing increased gamma oscillation power, impaired recognition memory, and increased stereotypic behaviors. In contrast, male rats did not exhibit these changes, highlighting sex differences in the pharmacological effects of NMDA antagonists, which could inform future research into treatments for neuropsychiatric disorders like schizophrenia and depression.

In recent years, physical activity has also attracted increasing attention from researchers (Wang et al., 2022). However, it is still not fully understood how neural oscillations are influenced by different types of exercise. In our Research Topic, there are two relevant review papers that deserve our attention. Li et al. explores how physical exercise (PE), including both aerobic and resistance training, promotes brain plasticity through neural oscillations. PE enhances cognitive function by modulating brain activity, especially in frequency bands like delta, theta, alpha, beta, and gamma. Exercise increases neurotrophic factors like BDNF and IGF-1, which support brain structures like the hippocampus and prefrontal cortex. The review emphasizes how different types of exercise, such as mind-body practices, affect neural activity, offering therapeutic potential for age-related cognitive decline and neurodegenerative diseases. Peng et al. explores Beta-band corticomuscular coherence (Beta-CMC), which refers to the synchronization of brain and muscle activity during movement. Beta oscillations (12–30 Hz) are crucial for motor control, influencing movement planning and execution. The review discusses Beta-CMC's role in various conditions such as motor disorders, rehabilitation, and athletic performance. It highlights the mechanisms of Beta oscillations in the sensorimotor system and their clinical applications, particularly for neurofeedback and personalized neuromodulation, aiming to improve therapeutic and athletic outcomes.

In sum, this Research Topic provides valuable new insights into the complex mechanisms of neural oscillations in the context of mood disorders, animal models, and physical exercise. Although much remains to be understood, these studies contribute significantly to advancing our knowledge and offer promising therapeutic directions.
